# Granulocyte colony-stimulating factor (G-CSF) treatment in combination with transplantation of bone marrow cells is not superior to G-CSF treatment alone after cortical stroke in spontaneously hypertensive rats

**DOI:** 10.3389/fncel.2014.00411

**Published:** 2014-12-04

**Authors:** Kai Diederich, Antje Schmidt, Carolin Beuker, Jan-Kolja Strecker, Daniel-Christoph Wagner, Johannes Boltze, Wolf-Rüdiger Schäbitz, Jens Minnerup

**Affiliations:** ^1^Department of Neurology, University of MünsterMünster, Germany; ^2^Fraunhofer Institute for Cell Therapy and ImmunologyLeipzig, Germany; ^3^Translational Center for Regenerative Medicine, University of LeipzigLeipzig, Germany; ^4^Department of Neurology, EVK BielefeldBielefeld, Germany

**Keywords:** stroke, spontaneously hypertensive rat (SHR), bone marrow cells, G-CSF, neuroregeneration, neurogenesis, angiogenesis, functional recovery

## Abstract

Granulocyte-colony stimulating factor (G-CSF) and bone marrow derived mononuclear cells (BM-MNCs) have both been shown to improve functional outcome following experimental stroke. These effects are associated with increased angiogenesis and neurogenesis. In the present study, we aimed to determine synergistic effects of G-CSF and BM-NMC treatment on long-term structural and functional recovery after photothrombotic stroke. To model the etiology of stroke more closely, we used spontaneously hypertensive (SH) rats in our experiment. Bone marrow derived mononuclear cells transplantation was initiated 1 h after the onset of photothrombotic stroke. Repeated G-CSF treatment commenced immediately after BM-MNC treatment followed by daily injections for five consecutive days. The primary endpoint was functional outcome after ischemia. Secondary endpoints included analysis of neurogenesis and angiogenesis as well as determination of infarct size. Granulocyte-colony stimulating factor treated rats, either in combination with BM-MNC or alone showed improved somatosensory but not gross motor function following ischemia. No beneficial effect of BM-MNC monotherapy was found. Infarct volumes were comparable in all groups. In contrast to previous studies, which used healthy animals, post-stroke neurogenesis and angiogenesis were not enhanced by G-CSF. In conclusion, the combination of G-CSF and BM-MNC was not more effective than G-CSF alone. The reduced efficacy of G-CSF treatment and the absence of any beneficial effect of BM-MNC transplantation might be attributed to hypertension-related morbidity.

## Introduction

Stroke is a lethal disease, yet it disables more than it kills. Better controls of risk factors in preventing stroke and improved treatment options for those who have had a stroke are essential for diminishing its devastating consequences. There are substantial research efforts being made to develop treatments that improve the outcome following stroke. Both, the stimulation of endogenous bone marrow cells by the granulocyte colony-stimulating growth factor (G-CSF) and the transplantation of bone marrow mononuclear cells (BM-MNCs) were shown to enhance regeneration in a large number of animal stroke studies (Schneider et al., 2005; Giraldi-Guimarães et al., 2009; Diederich et al., 2012b). Mechanisms underlying G-CSF and BM-MNC induced functional recovery after stroke include the potentiation of endogenous neurogenesis and angiogenesis as well as an increased dendritic plasticity (Lee et al., 2005; Schneider et al., 2005). Granulocyte-colony stimulating factor mediates its actions on these mechanisms by the mobilization of bone marrow cells and by direct neuronal effects (Schneider et al., 2005). The BM-MNC mode of action is assumed to be based on their production and secretion of cytokines, which in turn modulate endogenous repair mechanisms. We hypothesized that the combination of G-CSF and BM-MNCs is more effective than either treatment alone because transplantation of exogenous BM-MNCs can bridge the gap until G-CSF mobilizes endogenous BM-MNCs into the blood. In addition, according to its genuine function, G-CSF might increase the survival of transplanted BM-MNCs and thereby improve their efficacy.

Besides its pro-regenerative effects G-CSF also exerts neuroprotective actions in animal stroke models (Minnerup et al., 2008; England et al., 2009; Sevimli et al., 2009; Strecker et al., 2010). These neuroprotective modes of action, however, could not be confirmed in a recent clinical trial (Ringelstein et al., 2013). In our study we focused on G-CSFś effects on regeneration rather than on neuroprotective actions. We used the photothrombotic stroke model, which is characterized by small and highly reproducible infarct sizes and the reliable evocation of neuroregenerative events like neurogenesis, angiogenesis and dendritogenesis (Carmichael, 2005). We chose spontaneously hypertensive (SH) rats for our experiments for two reasons: first, the use of healthy animals without comorbidities was demonstrated to overstate the efficacy of candidate stroke treatments and second, SH rats are syngeneic, thus transplantation of BM-MNCs from one SH rat to another simulates autologous transplantation, which might be more practical from a translational perspective.

## Experimental procedures

### Animals

All animal procedures were approved by the responsible ethics committee of the University of Münster and the appropriate authorities of the Federal State of North Rhine-Westphalia. The investigations were carried out in accordance with national and international animal welfare regulations and are reported in accordance with the Animal Research: Reporting *In Vivo* Experiments (ARRIVE) guidelines (Kilkenny et al., 2011). Surgery and evaluation of all read-outs were performed blinded to experimental groups. Experiments were performed on adult (12–13 weeks old) male SH rats weighing 260–290 g. Spontaneously hypertensive rats were shown to have an increased blood pressure starting from 5 to 6 weeks of age (Dickhout and Lee, 1998). All animals were randomly assigned to one of the following treatment groups: (1) placebo (*n* = 13); (2) G-CSF 50 µg/kg/day (*n* = 14); (3) 5 million BM-MNCs/rat (*n* = 14); or (4) 5 million BM-MNCs/rat and G-CSF 50 µg/kg/day (*n* = 14). One animal of the placebo group died during surgery. BM-MNC transplantation was initiated 1 h after the onset of the photothrombosis. Repeated G-CSF treatment started immediately after BM-MNC treatment followed by daily injections for five consecutive days.

The cell numbers used in our experiments were based on previous studies that investigated different intravenous cell therapies in animal stroke models (Iihoshi et al., 2004; Giraldi-Guimarães et al., 2009; Minnerup et al., 2014). The condition of animals was monitored at least every 8 h. Pre-defined termination criteria were: (1) a severe immobility; and (2) a persisting abnormal body position. The implementation of these criteria was required by the local ethics committee.

### Bone marrow mononuclear cell preparation

Bone marrow derived mononuclear cells were prepared as previously described (Minnerup et al., 2014). Briefly, syngeneic rat bone marrow was obtained from male SH rats at the age of 12 weeks. Femurs and tibias were aseptically opened and repeatedly flushed with phosphate buffered saline (PBS). After erythrocyte lysis by ammonium chloride-based buffer (0.155 M NH_4_Cl, 10 mM KHCO_3_ and 0.01 mM Na_2_EDTA) cells were filtered by a 100 µm cell strainer, counted and prepared for immunomagnetic depletion of granulocytes: bone marrow cells were incubated with 10 ng/ml Phycoerythrin-conjugated anti-rat granulocyte antibody (clone RP1; BD Pharmingen, Heidelberg, Germany) for 15 min at 4°C. Subsequently, cells were washed with cold PBS plus 0.5% fetal calf serum (FCS) and incubated with 200 µl anti-Phycoerythrin MicroBeads (Miltenyi Biotech, Bergisch Gladbach, Germany) in 800 µl PBS plus 5% FCS for 15 min at 4°C. After incubation, non-adsorbed MicroBeads were removed by a further washing step. The cell suspension was then resuspended in 500 µl PBS plus 0.5% FCS and magnetically separated by a LD-column according to the manufacturer’s instructions (Miltenyi). This procedure results in higher BM-MNC purity compared to standard density gradient centrifugation (Pösel et al., 2012). The obtained mononuclear cell fraction was collected, counted, cryopreserved in liquid nitrogen (25 million mononuclear cells in 1 ml FCS plus 8% DMSO) and stored at −80°C until further use. Vital cell numbers were determined by the trypan blue exclusion method using a hemocytometer (Pösel et al., 2012). Cellular composition of cell grafts was characterized by flow cytometry for B cells (CD45R+), T cells (CD3+) and myeloid cells (CD11b+ and RP1-).

### Stroke model and therapy

The photothrombotic stroke model was utilized in this study and was executed as previously described (Schmidt et al., 2012). In brief, animals were anesthetized with an intraperitoneal injection of ketamine hydrochloride (100 mg/kg body weight; Ketanest) and xylazine hydrochloride (8 mg/kg body weight). The left femoral vein was cannulated with a PE-50 tube for Bengal Rose infusion. The rectal temperature was maintained at 37°C by a thermostat-controlled heating pad (Föhr Medical Instruments). Photothrombotic ischemia was induced in the right frontal cortex. For illumination, a laser spot of 8 mm in diameter (G Laser Technologies) was placed stereotaxically onto the skull 0.5 mm anterior to the bregma and 3.5 mm lateral from the midline. The skull was illuminated for 20 min. During the first 2 min of illumination, the dye Bengal Rose (0.133 mL/kg body weight, 10 mg/mL saline) was injected intravenously. One hour after onset of the photothrombosis animals received BM-MNCs or vehicle intravenously. Animals of the respective treatment groups received treatment with G-CSF or saline daily for five consecutive days starting immediately after BM-NMC-treatment. To label dividing cells, each animal received a daily bromodeoxyuridine (BrdU) injection (50 mg/kg per day intraperitoneally) throughout the 5-day treatment period, 1 h before the respective G-CSF or saline injection.

### Functional testing

Behavioral testing was conducted as previously described (Diederich et al., 2012a). In all animals, behavioral tests were performed before ischemia (baseline) as well as on days 1, 7, 14, 21, and 28 after ischemia by an investigator blinded to the experimental groups.

Motor deficits were examined by means of the cylinder test. For this purpose, the rats were placed into a transparent cylinder and videotaped from below for 3 min. Spontaneous wall and ground touches of the forepaws were counted. An asymmetry score calculated for each animal was expressed by the following ratio: wall and ground touches of the ipsilateral forepaw—wall and ground touches of the contralateral forepaw/wall and ground touches of the ipsilateral forepaw+wall and ground touches of the contralateral forepaw.

Somatosensory deficits were measured using the adhesive removal test. Two small pieces of adhesive-backed paper dots of equal size used as bilateral tactile stimuli occupying the distal–radial region were placed at the wrist of each forelimb. The time to remove each stimulus was documented. An asymmetry score calculated for each animal was expressed by the following ratio: time to remove the ipsilateral dot—time to remove the contralateral dot/time to remove the ipsilateral dot+time to remove the contralateral dot.

### Tissue processing

Twenty-eight days after ischemia, animals were anesthetized and transcardially perfused with 4% paraformaldehyde in 0.1 mol/L phosphate buffer. The brains were fixed in 4% paraformaldehyde at 4°C and then cryoprotected in 30% sucrose solution. Tissue was stored at −80°C until analysis.

### Determination of infarct size

Lesion volumes were estimated by measurement of the maximum diameter and measurement of the maximum infarct areas on the slides, as previously described (Müller et al., 2008). Because infarct size and tissue loss do not always match, an additional analysis of the remaining cortical tissue was performed at the level of the largest infarct extension.

### Immunohistochemistry

Immunohistochemistry was performed on coronal free-floating 40 µm sections with the following antibodies: anti-NeuN (1:200; Millipore, Darmstadt, Germany), rat anti-BrdU (1:500; Abcam, Cambridge, UK), mouse anti-neuronal nuclei and rat anti-CD31 (1:250; Abcam), anti-Iba1 (raised in goat, 1:200, Abcam, Cambridge, UK). Detection of anti-NeuN antibodies was done with a goat anti-mouse fluorescent dye (AlexaFluor488, 1:100, 45 min.; MolecularProbes, Leiden, Netherlands). Bromodeoxyuridine antibodies were detected using a biotin conjugated goat-anti-rat antibody (1:500; 45 min; Jackson Labs, West Grove, PA, USA); CD31-antibodies detection was done using a biotin conjugated goat-anti-rat antibody (1:100; 45 min; Jackson Labs). Detection of Iba1-antibodies was performed using a biotin conjugated donkey anti-goat antibody (1:200, 45 min, room temperature, Jackson Labs, West Grove, PA, USA).

For signal amplification of BrdU-, CD31- and Iba1-signal, sections were incubated with horseradish peroxidase/streptavidin (1:100, 45 min; DAKO, Glostrup, Denmark) and biotinyl tyramide (1:100). Bromodeoxyuridine, CD31- and Iba1-positive cells were visualized by a streptavidin/fluorescent dye (AlexaFluor594, Molecular Probes). Nuclei counterstain was done with a tissue preserving medium containing 4′,6-diamidino-2-phenylindole (DAPI, Vector, Burlingame, CA, USA). Immunoflurescence was computed and visualized with a Nikon Eclipse 80i fluorescence microscope (Nikon, Düsseldorf, Germany) equipped with proper filter sets for AlexaFluor594, AlexaFluor488 and DAPI.

### Analysis of the cellular inflammatory response

Quantification of Iba1-positive cells was performed by counting absolute cell amounts covering four random squares (200 × 200 µm) within the ipsilateral boundary zone of the infarct of four separate brain sections per animal.

### Analysis of neurogenesis

Quantification of neurogenesis was performed as described previously (Diederich et al., 2012a). Briefly, BrdU/NeuN-positive cells were analyzed in three brain regions: the dentate gyrus (DG), subventricular zone (SVZ), and peri-infarct area (PI). In the DG and SVZ, all BrdU-positive cells were counted on seven sections (every 12th section, 440-µm intervals) per hemisphere. For the analysis of BrdU/NeuN-positive cells in the PI, four squares (300 µm × 300 µm) adjacent to the PI were analyzed on four sections (bregma 1 mm to −0.5 mm). To determine the percentage of neurons among the newly generated cells, 50 randomly selected BrdU-positive cells within the DG, SVZ, and PI, respectively, were analyzed for BrdU/NeuN co-labeling. Multiplying the total number of BrdU-positive cells with the percentage of NeuN/BrdU, double-positive cells yielded the number of new neurons in the respective areas.

#### Analysis of angiogenesis

Vessel length was determined by calculating the area of CD31 staining using ImageJ v1.34 software program (NIH). Three areas (20× magnification) depicting the ischemic border zone from three sections per animal, respectively, were photographed and subsequently analyzed. Vessel cross-sectional surface area and perimeter were measured using Neurolucida (MicroBrightField). Two vessels featuring a minimum length of 300 µm for two sections were analyzed per animal.

#### Primary and secondary objectives of the study

According to the ARRIVE guidelines primary and secondary objectives were defined (Kilkenny et al., 2010). The primary endpoint was functional outcome after ischemia. Secondary endpoints were the structural outcome as analyzed by the generation of new neurons and angiogenesis as well as infarct size.

#### Statistical analysis

Randomization was carried out by the computer software “Research Randomizer” (Version 3.0; Urbaniak GC, Plous S, 2011, retrieved on March 23, 2011, from www.randomizer.org/). The values presented in this study are means ± SEM. Statistical analyses were calculated using the Statistical Package of Social Sciences (Version 15.0; SPSS Inc., Chicago, IL, USA). The normality distribution of the data was assessed by graphical examination of the histograms and verified by the Shapiro-Wilk test (*P* > 0.05). Behavioral measurements were analyzed by area under the curve analysis using analysis of variance (ANOVA) followed by the Fisher protected least significant difference test. Student *t*-test with Bonferroni correction was used to compare data between two groups. An α error rate of 0.05 was taken as the criterion for significance.

## Results

All experiments were performed on a total number of 55 animals (placebo: *n* = 13, G-CSF: *n* = 14, BM-MNC: *n* = 4, BM-MNC and G-CSF: *n* = 14).

### Functional outcome

**Figure 1:**
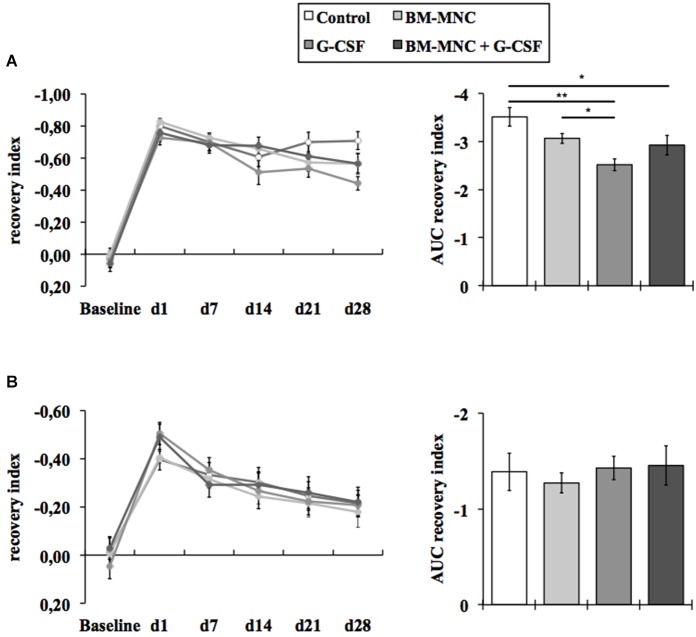
**Assessment of neurological deficits**. Somatosensory recovery was assessed by the adhesive removal test **(A)**. Animals of all experimental groups exhibited notable deficits in somatosensory function following photothrombotic stroke, which subsequently attenuate over the course of 28 days until the end of the experiment. The area under the curve (AUC) analysis revealed a significantly improved functional recovery after G-CSF monotherapy and G-CSF + BM-MNC combination (*P < 0.05, **p < 0.01; Fisher protected least significant difference *post hoc* test after significant ANOVA). Motor recovery was assessed by the cylinder test **(B)**. Animals of all experimental groups showed deficits in motor function, which attenuate over the course of 28 days until the end of the experiment. The AUC analysis of the cylinder test did not reveal any significant treatment effects on the recovery of motor functions (*P* = 0.859; one-way ANOVA).

The photothrombotic stroke model causes distinct deficits in somatosensory and motor functions. Somatosensory recovery was evaluated by the adhesive removal test (Figure 1A) and motor recovery by means of the cylinder test (Figure 1B). A summarized analysis of the functional recovery was performed by means of the area under the curve. Baseline performance was comparable between all treatment groups. As expected, animals of all experimental groups exhibited notable deficits in somatosensory and motor function following photothrombotic stroke, which subsequently attenuate over the course of 28 days until the end of the experiment (Figures 1A,B).

The area under the curve analysis of the adhesive removal test revealed a statistically significant decrease of the somatosensory deficit in G-CSF (*P* < 0.01, Fisher protected least significant difference *post hoc* test after significant ANOVA, Figure 1A) and in the G-CSF+BM-MNC (*P* < 0.05) treated animals compared to vehicle treated animals. In addition, G-CSF treated animals also displayed a significantly improved somatosensory recovery compared to animals treated with BM-MNC alone (*P* < 0.05).

The area under the curve analysis of the cylinder test did not reveal any significant treatment effects on the recovery of motor functions (*P* = 0.859; one-way ANOVA, Figure 1B).

### Infarct volumes

**Figure 2:**
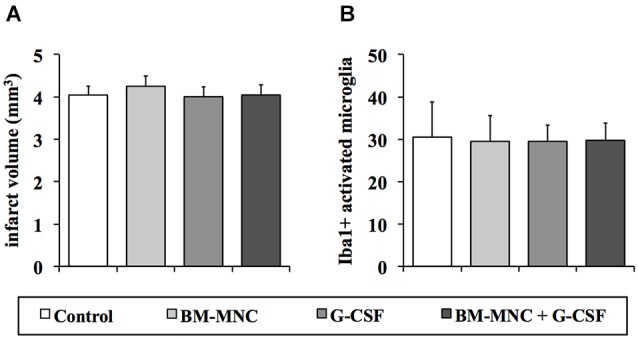
**Analyses of infarct volumes and the postischemic inflammatory response 28 days after photothrombotic stroke**. Infarct volumes **(A)** did not differ between the treatment groups (*P* = 0.877; one-way ANOVA). There was no statistical difference between the groups regarding the number of activated microglia cells (Iba1-positive, **(B)**) in the boundary zone of the infarct (*P* = 0.976; one-way ANOVA).

As expected after photothrombotic stroke, infarct volumes did not differ between the four groups (*P* = 0.877; ANOVA, Figure 2A).

### Analysis postischemic inflammation

To investigate treatment effects on postischemic inflammation in the chronic phase following ischemia we analyzed the extent of the inflammatory response 28 days after infarct induction. The number of Iba1-positive cells with retracted processes and round cell bodies, representing activated microglia, did not differ between the different groups (*P* = 0.976; one-way ANOVA, Figure 2B).

### Quantification of postischemic neurogenesis

**Figure 3:**
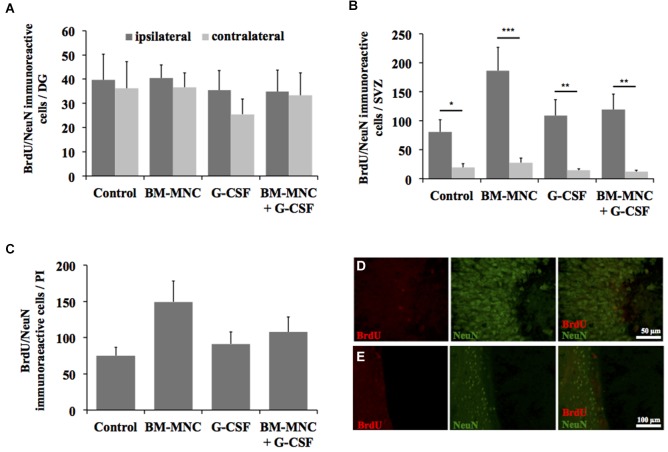
**Analysis of post-stroke neurogenesis**. Quantification of neurogenesis by detection of BrdU/NeuN-expressing cells in the dentate gyrus (DG, **A**), subventicular zone (SVZ, **B**), and peri-infarct area (PI, **C**) (*P < 0.05, **P < 0.01, ***P < 0.001; Student *t*-test with Bonferroni correction). Representative photomicrographs from the DG **(D)** and SVZ **(E)** demonstrating BrdU/NeuN-expressing cells.

The analysis of postischemic neurogenesis revealed no significant treatment effects on the generation of new neurons in any of the analyzed regions in postischemic brains of SH animals (Figures 3A–E). As expected, the amount of BrdU/NeuN immunoreactive cells was significantly elevated in the SVZ of the ipsilateral compared to the contralateral hemisphere in animals of all treatment groups (ipsilateral vs. contralateral, control group: *P* < 0.05; BM-MNC group: *P* < 0.001; G-CSF group: *P* < 0.01; BM-MNC+G-CSF group: *P* < 0.01, Students *t*- test with Bonferroni correction, Figure 3B). A comparison of the treatment groups did not reveal significant treatment effects on the amount of newborn neurons in the SVZ in either of the two hemispheres (ipsilateral: *P* = 0.101, contralateral: *P* = 0.108; multivariate ANOVA, Figure 3B). Furthermore, no treatment effects on postischemic neurogenesis were detected in the DG and in the PI (DG ipsilateral: *P* = 0.948, contralateral: *P* = 0.743; ANOVA, Figure 3A/PI *P* = 0.095; one-way ANOVA, Figure 3C). In the DG, no differences were found between the contralateral and ipsilateral hemisphere (ipsilateral vs. contralateral control group: *P* = 0.830; BM-MNC group: *P* = 0.637; G-CSF group: *P* = 0.349; BM-MNC+G-CSF group: *P* = 0.907, Students *t*-test with Bonferroni correction, Figure 3A).

### Quantification of postischemic angiogenesis

**Figure 4:**
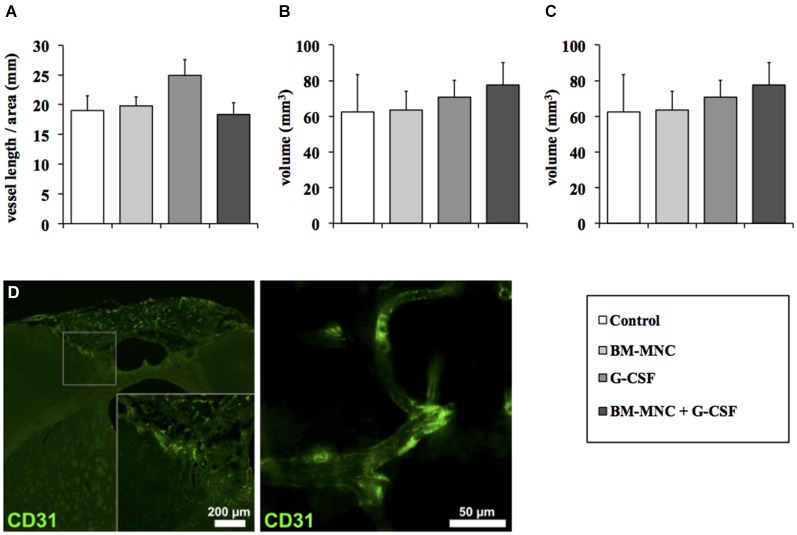
**Analysis of post-stroke angiogenesis**. Quantification of total vascular length per area **(A)**. The vessel length per area did not differ between the groups (*P* = 0.236; ANOVA). Analysis of vessel surface area **(B)** and perimeter **(C)**. No qualitative changes in surface area (*P* = 0.949; ANOVA) and volume (*P* = 0.848; ANOVA) were detected. **(D)** Representative photomicrographs demonstrating blood vessels (CD31) within the peri-infarct region.

To investigate effects of the different treatment groups on angiogenesis (Figures 4A–D), blood vessel length was assessed at the ischemic border zone. There was no difference in vessel length between the groups (*P* = 0.236, one-way ANOVA, Figure 4A). In addition, analysis of individual vessels revealed no qualitative changes in surface area (*P* = 0.949, one-way ANOVA, Figure 4B) and volume (*P* = 0.848, one-way ANOVA, Figure 4C).

## Discussion

The present study investigated the hypothesis that the combination of G-CSF and BM-MNCs is more effective than either treatment alone. Granulocyte-colony stimulating factor treated rats, either in combination with BM-MNCs or alone, showed a significantly improved functional recovery compared to placebo rats measured by the adhesive removal test. In contrast, early BM-MNC monotherapy did not improve somatosensory rehabilitation. Regeneration of gross motor function as tested by the cylinder test remained unaffected in animals of all treatment groups. The observed improvement of functional recovery in G-CSF-treated animals was not due to infarct volume reduction, thus suggesting a true recovery-enhancing effect of G-CSF rather than neuroprotective actions. Post-stroke neurogenesis and angiogenesis were not significantly enhanced after G-CSF treatment. Furthermore, the extent of the local inflammatory response to cerebral ischemia were comparable in all treatment groups.

Initial reports indicated that treatment with BM-MNC might reduce neurological impairment in animal models of focal cerebral ischemia after application in acute and subacute phases (Iihoshi et al., 2004; Kamiya et al., 2008; Brenneman et al., 2009; Giraldi-Guimarães et al., 2009; Nakano-Doi et al., 2010; Kasam et al., 2012). However, there is cumulating evidence, that the efficacy and therapeutic applicability of BM-MNC-treatment might be more limited than initially anticipated. In contrast to previous studies which used healthy animals, a recently published study from our group could not demonstrate beneficial effects of early BM-MNC treatment following transient middle cerebral artery occlusion (MCAO) in SH animals. Infarct volumes, early behavioral outcomes, and the extent of the immediate inflammatory response to cerebral ischemia were not affected by BM-MNC treatment in co-morbid animals (Minnerup et al., 2014).

In addition, timing of cell administration may have limited the therapeutic impact of BM-MNC transplantation as a therapeutic efficacy of BM-MNC application was previously shown for a time window between 3 h and 7 days only (Iihoshi et al., 2004; Vasconcelos-dos-Santos et al., 2012). Since we found that BM-MNC in combination with G-CSF treatment time-dependently abolished the beneficial effect of G-CSF on long-term functional rehabilitation after MCAO in co-morbid SH animals (Pösel et al., 2014), we chose 1 h as the time point for BM-MNC application to reduce the likeliness for such detrimental interaction. Whether this very early timing has contributed to the lack of therapeutic impact remains for further investigation. However, the most important difference in our study design is the use of co-morbid animals as opposed to healthy animals. In fact, divergent results on effect size with greater infarct size reductions in healthy animals compared to animals with comorbidities were previously reported (Crossley et al., 2008). The predominant use of young and healthy animals in preclinical studies is assumed to contribute considerably to the translational failure in experimental stroke research (Crossley et al., 2008).

While the efficacy of BM-MNC treatment on post-stroke regeneration is diminished by the occurrence of co-morbidities, the rehabilitative effects of G-CSF are still ascertainable, albeit seemingly less pronounced. Zhao et al. (2007b) reported limited and unstable long-term functional improvement following MCAO in SH rats when G-CSF was administered daily, 3 h to 7 days after ischemia. When G-CSF treatment was delayed until 3.5 month after MCAO no functional benefit could be ascertained (Zhao et al., 2007a). In accordance with these findings, we detected a limited efficacy of G-CSF treatment following photothrombotic stroke in SH rats. While long-term somatosensory function was improved, as revealed by the adhesive removal test, the cylinder test did not detect any differences in motor function. In contrast, we recently demonstrated, that G-CSF monotherapy led to a robust improvement of both qualities following photothrombotic stroke in normotensive rats of the same age (Diederich et al., 2012b).

Granulocyte-colony stimulating factor treatment as well as transplantation of BM-MNC both potentiate angiogenesis and neurogenesis (Lee et al., 2005; Schneider et al., 2005; Giraldi-Guimarães et al., 2009; Nakano-Doi et al., 2010) after ischemia in normotensive animals. These mechanisms are suggested to be primarily mediating long-term neurorehabilitation in non-comorbid animals (Kojima et al., 2010; Osman et al., 2011). Increased neurogenesis (Kronenberg et al., 2007) and angiogenesis (Yang et al., 2011) occur in SH animals, regarded as part of a counter-regulatory repair process against hypertension-induced brain damage. Our results demonstrate, that in SH animals, these repair mechanisms are not enhanced by G-CSF-treatment following stroke and therefore do not mediate G-CSF-induced functional improvement. These findings indicate, that therapeutic modulation of angiogenesis and neurogenesis may be crucially limited by comorbid disease, which might result in reduced effectiveness. A potential explanation therefore might be an exhausted angiogenic and neurogenic reserve, as suggested for neurodegenerative diseases (Kempermann, 2008).

Transplanted BM-MNCs (Brenneman et al., 2009) as well as G-CSF (Sehara et al., 2007; Solaroglu et al., 2009; Dietel et al., 2012) exert neuroprotective anti-inflammatory actions after cerebral ischemia in normotensive animals. In SH animals, however, BM-MNC treatment did not affect the local microglial immune response after MCAO (Minnerup et al., 2014). In the current study, we investigated treatment effects on postischemic local inflammation in the chronic phase 28 days after photothrombotic stroke. Neither the respective monotherapies with BM-MNCs and G-CSF, nor the combination of both resulted in an altered local microglial response. This result indicates, that local inflammation might not be directly responsible for improved long-term functional recovery following G-CSF treatment. However, hypertension has been shown to entail systemic vascular inflammation including chronic activation of microglia (Zubcevic et al., 2011) and may therefore influence poststroke immune responses (Möller et al., 2014) and might also interfere with the therapeutic potential of G-CSF and BM-MNC.

Our study has strength and limitations. The experiments were conducted in concordance with recommendations for good preclinical stroke research (Sutherland et al., 2012) and rigorously adhered to stringent quality criteria in experimental stroke research such as randomization, surgery and evaluations performed in a blinded fashion, and controlled physiological parameters. Furthermore, the use of animals with a relevant comorbidity may increase the predictive value of the presented findings regarding a human stroke patient population. While we could demonstrate G-CSF-induced improvements in long-term functional recovery following cortical ischemia in co-morbid animals, the underlying mechanism remains to be determined. Analyses of post-stroke angiogenesis might be confounded by hypertension-related endothelial abnormalities. Further research is needed to determine how co-morbidities like hypertension affect post-stroke repair mechanisms. The results of the present study cannot be attributed to the co-morbid condition of the animals with absolute certainty, since no control groups of healthy animals were included in the present study. Future preclinical stroke studies on co-morbid animals should also include groups of healthy animals in order to determine whether the obtained results can be directly attributed to the comorbid condition. In the present study, we focused on syngenic BM-MNC transplantation as it simulates autologous transplantation. Bone marrow derived mononuclear cells derived from normotensive rats might differ in their phenotype and thus in their therapeutic capacity as compared to BM-MNC derived from hypertensive rats used in the present study. Further research is needed to determine the therapeutic potential of BM-MNCs and their limitations, including analysis of their cytokine profiles. The cell numbers used in our experiments were based on previous studies that investigated different intravenous cell therapies in animal stroke models (Minnerup et al., 2011). However, we cannot rule out that the use of a higher BM-MNC dose might have yielded different results.

Our study confirms the beneficial effect of G-CSF treatment on long-term functional recovery following cortical stroke in hypertensive animals. Angiogenesis and neurogenesis, which are regarded as decisive mechanisms of G-CSF-mediated regeneration in normotensive animals, remained unaffected by G-CSF treatment in hypertensive animals. Contrary to our hypothesis, the combination of G-CSF and BM-MNC was not more effective than G-CSF alone and the monotherapy with BM-MNC was without any effect on functional recovery. The reduced efficacy of G-CSF treatment and the absence of any beneficial effect of BM-MNC transplantation might be attributed to hypertension-related morbidity. The findings of our study further corroborate the importance of evaluating the efficacy of treatments and determining the underlying neuroregenerative mechanisms in animal models more adequately reflecting the characteristic pathophysiological state of stroke patients.

## Conflict of interest statement

Wolf-Rüdiger Schäbitz is an inventor on a patent claiming the use of granulocyte colony-stimulating factor for the treatment of stroke. The other authors report no conflicts.
